# Effects of fasting on the interplay between temperature and *Trypanosoma cruzi* infection on the life cycle of the Chagas disease vector *Rhodnius prolixus*

**DOI:** 10.1371/journal.pntd.0012665

**Published:** 2024-11-11

**Authors:** Henri Loshouarn, Alessandra A. Guarneri

**Affiliations:** Vector Behavior and Pathogen Interaction Group, Instituto René Rachou, Fundação Oswaldo Cruz-FIOCRUZ, Belo Horizonte, Brazil; Kenya Agricultural and Livestock Research Organization, KENYA

## Abstract

*Rhodnius prolixus*, a triatomine insect, is one of the most important vectors of Chagas disease in South America. Its interaction with the parasite *Trypanosoma cruzi*, the causative agent of this disease, is known to be deeply affected by ambient temperature and the nutritional status of the insect vector. In this study, we investigated how starvation affects the life cycle of *R*. *prolixus* and the population dynamics of the parasite inside the intestine of the vector at four temperatures ranging from 24°C to 30°C. The weights and molting times of chronically infected and uninfected insects were monitored through repeated 30-day fasting periods from first instar to adult stage, assessing their capacity to retain blood meal weight between developmental stages and tracking parasite concentrations in their urine. Our results demonstrate that ambient temperature is a crucial factor affecting the resistance of *R*. *prolixus* to starvation, as survival, body weight, and weight retention greatly decreased in high temperature treatments. Furthermore, we showed that temperature significantly influenced whether *T*. *cruzi* established an infection in early instars, with few insects developing infections at the lowest and highest temperature treatments. Additionally, we discovered that a fasting period of 30 days induces a steady decrease in parasite populations in the vector over its lifetime. Infection by *T*. *cruzi* had no effect on the survival, molting time, and nutritional factors monitored in our protocol. Our results highlight the importance of nutrition as a determining factor for the development of the vector and the parasite, providing valuable insights for elucidating the complex interplay between temperature and nutrition in shaping the epidemiology of Chagas disease in a changing climate.

## Author summary

In this study we explored how starvation and temperature affect the relationship between the insect *Rhodnius prolixus* and the parasite *Trypanosoma cruzi*, causative agent of Chagas disease. *R*. *prolixus* is a key vector for this disease in South America. We examined the impact of repeated 30-day fasting periods on the life cycle of the insect and the population of the parasite at temperatures ranging from 24°C to 30°C.

Higher temperatures negatively affected the survival of these insects under conditions of starvation. The insects lost more weight and had lower survival rates at higher temperatures. We also discovered that at 24°C and 30°C, fewer insects developed a chronic *T*. *cruzi* infection. Additionally, starvation led to a gradual reduction of the parasite population size in the insects over developmental stages. In this context, we found no effect of infection on *R*. *prolixus*.

Our findings highlight the crucial role of the vector nutritional status in the development and survival of both the insect and the parasite. This research deepens our understanding of how temperature and the nutrition status of the vector together influence the spread of Chagas disease, providing valuable insights for predicting the epidemiology of the disease in a changing climate.

## Introduction

*Rhodnius prolixus* is a hematophagous triatomine insect and the principal vector species involved in the domestic transmission of Chagas disease in the Andean Community region [1]. This parasitic infection occurs primarily within Latin American regions where it represents a significant public health challenge [2]. The causative agent of this disease, the protozoan *Trypanosoma cruzi*, is an obligatory parasite that infects both mammals and triatomines, which can function as host reservoirs and/or vector for the parasite. *T*. *cruzi* transmission usually occurs through contact with the insect vector, emphasizing the importance of understanding the ecological and biological factors influencing *R*. *prolixus* and its relationship with *T*. *cruzi* [3].

Triatomines acquire *T*. *cruzi* while feeding on an infected mammal, as every developmental stage of these insects feed on blood. Parasite development within the insect is restricted to the intestinal tract, where the rectal ampulla serves as the principal site for the production of infective trypanosome forms [4]. The presence of the parasite can cause an array of symptoms to the insect vector. The extent of these symptoms depends on both abiotic environmental and biotic factors, such as the physiological state of the triatomine, ambient temperature, parasite strain, and/or coinfection with other microorganisms [5–10]. Notably, *T*. *cruzi* infection impacts the resource allocation and/or amount of the vector, as *T*. *cruzi*-infected triatomines tend to have a lower fertility and ability to maintain body weight over time [8,11,12]. Infected insects also display increased mortality rates from 30 days post-blood feeding onward, and their survival is more heavily impacted by fasting than that of uninfected insects [7,12–14].

In turn, the nutritional state and habits of the triatomine host also influence the development of the parasite and the outcome of infection, as larger blood meals lead to an increase in parasite load at the next developmental stage of the triatomine [11], and a 90-day starvation period is enough to kill 99.5% of parasites in the rectal ampulla of fifth instar nymphs [4], which may result in the loss of infection [15]. In a previous study, we showed that in the context of chronic infection, the *T*. *cruzi* parasite load increases over developmental stages when the insect is maintained under 15-day fasting periods after molting [11]. However, there has been limited research into the dynamics of parasite load under varying triatomine nutritional statuses, even though the quantity of nutritional resources available to the parasite are thought to be among the most important limiting factors of parasite population growth [16].

The environmental temperature that the insect is exposed to is another important factor influencing the development of the parasite in the vector and the symptoms associated with infection, while also profoundly influencing the physiology, behavior, development, immunity and metabolic rates of the triatomine vector [7,11,17–20]. The presence of detrimental effects of infection such as higher mortality rates and molting delays is temperature-dependent [6,11,21]. Furthermore, *T*. *cruzi* development, multiplication rate, and first appearance in the feces of the vector are made possible and accelerated by higher temperatures [4,6,11,14,22,23]. The effects of temperature on *R*. *prolixus* could result in a significantly quicker vector reproductive cycle in the context of global warming, potentially leading to substantial consequences on vector populations and Chagas disease epidemiology [24,25]. Conversely, high temperatures also increase insect mortality, water loss rate, and depletion of the nutritional resources of the insect [7,11,18,26,27]. Deciphering these complex and paradoxical effects of temperature is particularly relevant because triatomine distribution and shelter choice have been shown to be tightly linked with temperature [28–30], and global warming has been established as the most significant climate change factor affecting insect species [31–33].

In this study, we investigated the impact of the fasting time of *R*. *prolixus* on the *T*. *cruzi* parasite load by monitoring the parasite concentration in the feces of triatomines subjected to 30-day starvation periods between blood meals. Additionally, *R*. *prolixus* mortality, intermolt period, and weight across developmental stages were recorded. This experiment was conducted at four temperatures ranging from 24 to 30°C, enabling us to explore the intricate relationships between *R*. *prolixus* nutrition, temperature, and *T*. *cruzi* development in the vector.

## Materials and methods

### Ethics statement

All experiments using live animals were performed in accordance with the Fundação Oswaldo Cruz (FIOCRUZ) guidelines on animal experimentation and were approved by the Ethics Committee in Animal Experimentation (Comissão de Ética no Uso de Animais de Laboratório; CEUA/FIOCRUZ) under the approved protocol number LW 03/22. The protocol we used is from the Conselho Nacional de Controle de Experimentação Animal of the Ministério da Ciência, Tecnologia e Inovações (CONCEA/MCTI; http://www.cobea.org.br/), which is associated with the American Association for Animal Science (AAAS), the Federation of European Laboratory Animal Science Associations (FELASA), the International Council for Animal Science (ICLAS) and the Association for Assessment and Accreditation of Laboratory Animal Care International (AAALAC).

### Organisms

The *R*. *prolixus* colony used in this study originated from insects collected in Honduras in the 1990s. Insects were maintained by the Vector Behavior and Pathogen Interaction Group at the Instituto René Rachou. Experimental triatomines were fed on SWR/J mice anesthetized with intraperitoneal injections of a mixture of ketamine (150 mg/kg mg/kg; Cristália, Brazil) and xylazine (10 mg/kg; Bayer, Brazil).

*Trypanosoma* infection was performed using the *T*. *cruzi* strain Dm28c originally isolated from a naturally-infected *Didelphis marsupialis* [34]. Parasites were cultured *in vitro* by two weekly passages in liver-infusion tryptose (LIT) medium supplemented with 15% fetal bovine serum (FBS), 100 mg/ml streptomycin and 100 UI/ml penicillin [35]. To prevent loss of infectivity, parasites were subjected to cycles of triatomine-mouse transmission every fifteen weeks.

### Experimental procedures

The experimental design used in this study was performed following the protocol from [11], with modifications. Briefly, two SWR/J mice were infected through intraperitoneal inoculation with 200 μl of triatomine urine containing metacyclic trypomastigotes of *T*. *cruzi*. On the ninth day after inoculation, the mice were anesthetized and exposed to 15 day-old first instar nymphs of *R*. *prolixus* that all fed at the same time until they were fully engorged (*n* = 120). The mice parasitemia was assessed using Brenner’s method and was found to be 15.48 parasites per μL of blood on average [36]. The first instar nymphs from the infected group ingested a mean ± SE of 2.73 ± 0.14 μL of blood, leading to an estimated 42.26 parasites ingested per triatomine individual. The control group, consisting of 120 triatomines, underwent the same procedures while feeding on an uninfected mouse. Following this initial blood meal, the insects were randomly allocated to one of four thermally-isolated boxes where they were subsequently maintained at one of four different fixed temperatures for the duration of the experiment. These temperatures were as follows, presented as the mean ± SE: 24 ± 0.3°C, 26 ± 0.2°C, 28 ± 0.2°C, and 30 ± 0.2°C. Each box maintained a 45 ± 5% RH and was subjected to a 12:12 light-dark (LD) illumination cycle. Each box contained 60 insects, half of which were infected and half of which were uninfected. Each triatomine was assigned a unique code name, and within each thermally-isolated box, they were kept individually in plastic containers (4 cm diameter × 2 cm high) covered by a piece of cloth and containing a piece of filter paper with feces from fifth instar nymphs of the colony to ensure the presence of symbionts. There was no contact between the individual insects, and the data were collected individually until the end of the experiment. The triatomines were inspected daily for molting and survival.

At each subsequent developmental stage, the insects received a single blood meal on uninfected anesthetized mice 30 to 31 days after each molt. Each triatomine was offered such a blood meal once per developmental stage, with an additional offering on the following day if the insect was not observed feeding during the first instance. In each blood meal, the insects were allowed to blood-feed to their full capacity, with the end of feeding determined as the moment the insects folded their proboscis and started walking. To ensure individual tracking, the insects were fed while inside plastic containers, each containing one individual triatomine, and tagged with their unique code name. The containers were closed by a piece of cloth that was put in contact with the anesthetized mice, allowing the insects to feed. The body weight of each individual triatomine was recorded both immediately before and immediately after blood-feeding. Immediately after the post-blood-feeding weighing, the triatomines were placed head up vertically for 150 min in Eppendorf tubes for urine collection, for which period they stayed in a 27°C incubator. After this collection period, the insects were returned to their original experimental temperature boxes until their next blood meal at the subsequent developmental stage. The urine volume was measured by counting the contents of the Eppendorf tube with a 0.5–10 μL micropipette. Next, 10 μL of undiluted homogenized insect urine was loaded in a Neubauer chamber for determination of the parasite concentration under light microscopy.

Weighing the insects immediately before and immediately after blood-feeding allowed the derivation of several variables: (i) the quantity of blood ingested (weight after blood-feeding–weight before blood-feeding), (ii) the total blood ingested until imaginal molting (the sum of the weights of all the individual nymphal blood meals), (iii) the blood ingestion ratio, which is the number of times of their own unfed weight the insects ingested in blood (blood-fed weight / unfed weight), and (iv) the retention performance, which is the fraction of the blood meal weight retained until the subsequent blood meal in the subsequent developmental stage. Retention performance was calculated using the following formula:


$$\mathrm{Retention}\;\mathrm{Performance}=\frac{\left(\mathrm{unfed}\;\mathrm{weight}(\mathrm{instar}\;N)-\mathrm{unfed}\;\mathrm{weight}(\mathrm{instar}\;N-1)\right)}{\mathrm{weight}\;\mathrm{of}\;\mathrm{blood}\;\mathrm{ingested}\;\mathrm{at}\;\mathrm{instar}\;N-1}$$

Where instar N– 1 is the preceding instar at which the blood meal was consumed, and instar N is the current instar at which the blood meal from instar N– 1 was already metabolized.

Due to limitations in the precision of the weighing scale used, accurate measurements for unfed nymphs in the first nymphal instar were not achievable. When this number was needed, an average value was obtained by simultaneously weighing all the nymphs of each treatment group and dividing the combined weight by the number of nymphs weighed. All weight measurements were performed using a microanalytical balance (Shimadzu AUW220D; accuracy of ±0.01mg).

All the triatomines that reached the adult stage were sexed and blood-fed 30 days after imaginal molting, as described above for the nymphal instars. The experiment was ended 60 days after the blood meal of the adult triatomines. The experiment lasted 293 days from the first instar nymphs blood-feeding to 60 days after the last blood meal of the adults.

### Statistical analysis

The effects of developmental stage, temperature, infection status (i.e., uninfected versus infected treatments, in the 26°C and 28°C treatment groups), and their interaction on different growth- and metabolism-related variables–namely (i) unfed weight, (ii) blood ingestion ratio, (iii) molting time, and (iv) retention performance–and parasite concentration and urine production were analyzed using generalized estimation equations (GEEs). The mandatory time-step used in the models was the developmental stage of the insects. Measurements from insects that died during the experiment were incorporated into the GEE models, as GEE modeling is well suited for datasets where measurements might be missing for individual samples at certain time points. The GEEs were generated with additional explanatory variables and interaction terms when their potential effect was suspected or needed to be accounted for. As only fifth instar nymph and adult sex was determined, the sex of the triatomines was not included in these analyses as this information is affected by survivorship bias. A comprehensive range of GEE models assuming different distributions and covariance matrices were tested for each variable studied. The best of all models generated for each individual variable was selected based on the lowest quasi-likelihood information criterion (QIC). When a Gaussian distribution was assumed in the model, residual normality was confirmed through QQplot examination. Model fitting and QIC determination were performed using the *geepack* [37] and *gee* packages [38] in R (version 4.2.1) [39]. Each graphical representation presented in this article was generated using the *ggplot2* package [40]. The details of the structures and results of all the models generated can be found in the supporting information of this article (S1 Statistical models).

Differences in survival were evaluated using Cox proportional hazards regression models with temperature and infection treatment as factors. The effect of infection treatment in each individual temperature treatment was then assessed separately using the same method. Survival at the end of the experiment was compared at each temperature depending on fasting time by binomial testing, using data from a similar experiment with a shorter fasting period [11]. The differences in the total volume of blood ingested until imaginal molting were tested using Student’s t-tests, and differences in the time from the first alimentation at the first nymphal instar to imaginal molting were tested using Mann–Whitney U tests. In both cases, pairwise comparisons were made between the uninfected control groups from each temperature treatment to assess the effects of temperature, and the effects of infection were tested by comparing the infection treatments (i.e., uninfected versus infected) within each temperature treatment group separately.

We used the nlme package [41,42] to perform a linear mixed effects analysis of three response variables, namely: (i) the dynamics of the parasite concentration in released urine, including an independent analysis of the effect of fasting using the data collected in an earlier similar experiment [11], (ii) the effects of the quantity of blood ingested on the parasite concentration in the subsequent developmental stage, and (iii) the correlation between individual parasite concentrations at one developmental stage to the next, across developmental stages and at each temperature treatment separately. In each linear mixed effect model, the term of the fixed effect was the developmental stage of the triatomine, and the random effect was specified as the individual triatomine sample nested within its developmental stage. This ensures that the model accounts for the repeated measurement of the same individuals. Visual inspection of residual plots did not reveal any obvious deviations from homoscedasticity or normality.

All numerical values presented in the text alongside confidence intervals represent the means ± standard errors (SEs). In all the statistical tests performed in this study, the level of significance was set to α ≤ 0.05.

## Results

### Parasite concentration in excreted urine

The establishment of chronic infection by *T*. *cruzi* in the triatomines was different between temperature treatment groups. None of the 30 triatomines from the 30°C treatment group that were fed on the infected mouse at first instar exhibited detectable parasites in their urine at any developmental stage, and only three out of the 30 insects from the 24°C treatment group developed a detectable chronic infection, which lasted for the entire length of the experiment in all three individuals. The infection success rate was greater in the 26°C and 28°C temperature treatment groups, with 29 and 28 of the 30 triatomines, respectively, becoming chronically infected. Some triatomines seemingly lost their infection during their lifetime, as three individuals (one from the 26°C treatment group and two from the 28°C treatment group) did not exhibit any parasites in their released urine after the fifth nymphal instar, despite being infected at earlier instars.

The GEE model analyzing the effects of developmental stage, unfed body weight, and temperature treatment on the concentration of parasites in released urine in the triatomines from the 26°C and 28°C temperature groups showed an effect of developmental stage in both temperature groups (26°C: Wald statistic = 4.45 and p = 0.035; 28°C: Wald statistic = 6.4 and p = 0.011). In addition, in the 28°C treatment group, there was a significant effect of unfed body weight (Wald statistic = 15.8 and p = 7e-05), although linear regression models did not reveal a significant correlation between unfed body weight and parasite concentration at any studied developmental stage (i.e., at the third, fourth, fifth, and adult stages), suggesting that this result reflects a general trend across developmental stages rather than being confined to a particular life phase. This effect had a negative estimate of -0.0445 in the GEE model, which implies a negative correlation between the unfed body weight of the triatomines and the parasite concentration in their released urine.

**Fig 1:**
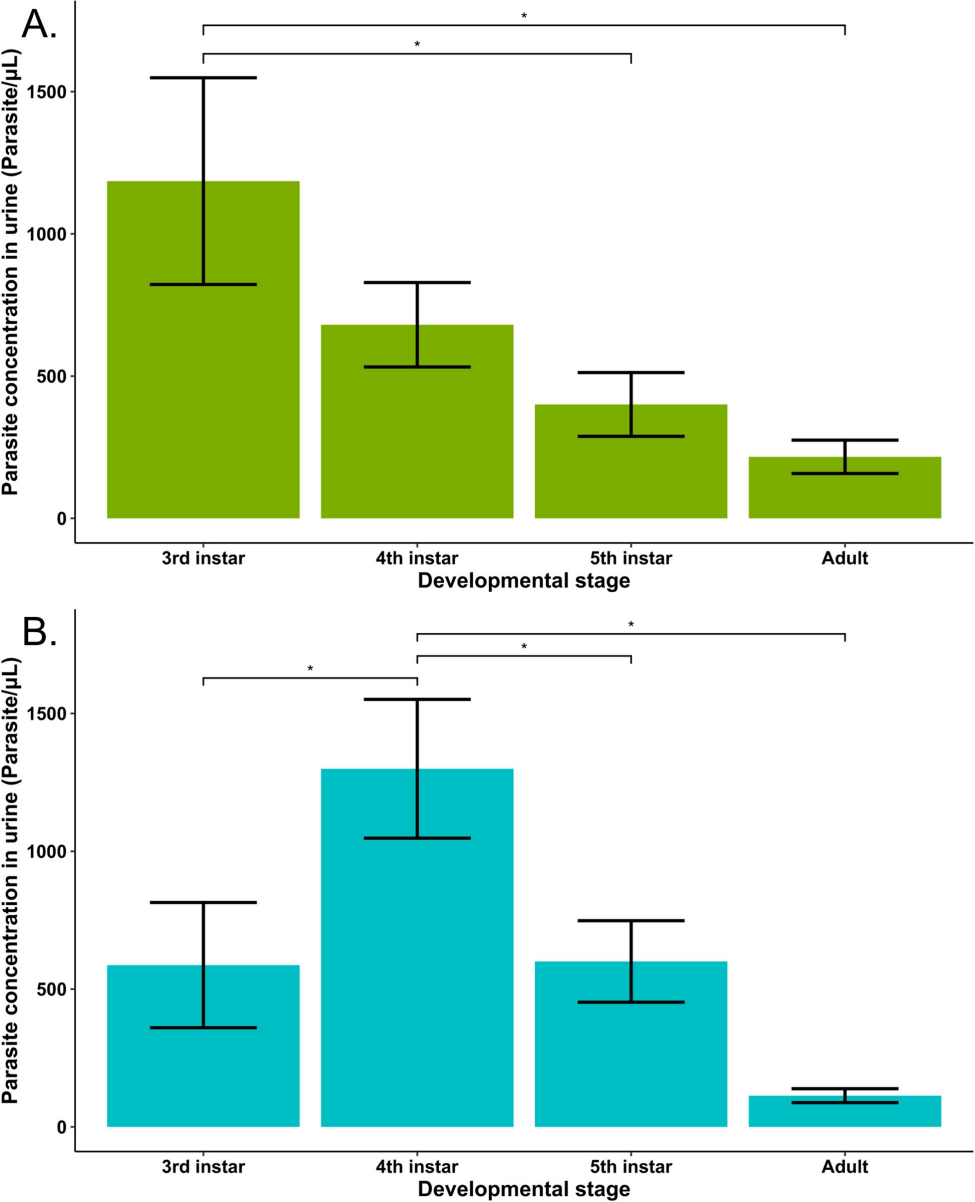
The mean parasite concentration in the excreted urine of *Trypanosoma cruzi*-infected *Rhodnius prolixus* maintained at (A) 26°C and (B) 28°C at different triatomine developmental stages. The differences between developmental stages were analyzed by linear mixed-effects modeling, taking the individual triatomine as random factor. (A) Third instar nymphs compared with: fifth instar (p = 0.0302); and adult stage (p = 0.0337) (n = 19). (B) Fourth instar compared with: third instar (p = 0.0351); fifth instar, (p = 0.0181); and adult stage (p = 0.0191) (n = 20). Error bars represent the standard error of the mean.

Since no effect of temperature treatment on the parasite concentration was observed, we conducted a linear mixed-effects model analysis on both temperature groups separately, investigating the differences in parasite concentration between developmental stages. The parasite concentration in the 26°C treatment group tended to decrease across successive developmental stages, going from 1185 ± 363 parasites per μL of urine at the third instar to 216 ± 58.6 at the adult stage. The linear mixed effect models for this temperature showed that the parasite concentration at the third instar was significantly higher when compared to the fifth instar (S.E. = 278.1, p = 0.0302 on 6 degrees of freedom) and the adult stage (S.E. = 353.7, p = 0.0337 on 6 degrees of freedom, n = 19), with no other significant differences between developmental stages (Fig 1A). For the 28°C treatment group, the parasite concentration peaked at 1299 ± 252 parasites per μL of urine in the fourth instar, before decreasing progressively in subsequent developmental stages. This resulted in a significantly greater parasite concentration in the fourth instar compared to every other developmental stage studied (comparison with: third instar, S.E. = 284.7, p = 0.0351 on 10 degrees of freedom; fifth instar, S.E. = 226.1, p = 0.0181 on 7 degrees of freedom; and adult stage, S.E. = 377.6, p = 0.0191 on 7 degrees of freedom, n = 20), with no other significant difference between developmental stages detected in our models (Fig 1B).

Multiple separate simple linear regression models were created to investigate how (i) the volume of blood ingested in the preceding developmental stage and (ii) the parasite concentration in released urine in the preceding developmental stage correlated with the parasite concentration at the analyzed developmental stage. This was performed for both the 26°C treatment group and the 28°C treatment group separately and for each developmental stage studied (namely, the third, fourth, fifth, and adult stages for the effect of the quantity of blood ingested in the preceding developmental stage and for the fourth, fifth, and adult stages for the correlation between parasite concentrations across developmental stages). No correlation was found between the quantity of blood ingested at the preceding developmental stage and the parasite concentration at any developmental stage at either of the two temperatures. As for parasite concentrations across developmental stages, the only significant correlation was that parasite concentrations between the fourth and fifth instars in triatomines kept at 28°C were positively correlated (*R*^*2*^ = 0.239, F _[__1__,__21__]_ = 6.58, p = 0.0180, n = 23), indicating that the parasite concentration carries over between these two instars at 28°C.

We compared the evolution of parasite concentration in insect urine with that from an earlier, similar protocol with 15 days of fasting after molting [11], and the linear mixed effect models shows a significant difference both in the 26°C (S.E. = 708, p = 0.00001 on 39 degrees of freedom, n = 41) and 28°C (S.E. = 755, p = 0.0062 on 36 degrees of freedom, n = 38) (S1 Fig). Pairwise Mann-Whitney U tests between the starved and non-starved triatomines at each developmental stage shows that this difference mainly arises from the parasite load being greater at third instar (p = 5.632e-06 and n = 60) and lower at adult stage (p = 0.01658 and n = 29) in starved insects.

### Life-cycle

GEE models were created simultaneously analyzing the effects of developmental stage, temperature and infection on the variables unfed body weight, blood ingestion ratio, retention performance, quantity of urine excreted and molting time, using only data from the 26°C and 28°C temperature groups, as they were the only ones in which infection rates allowed for statistical analysis. For each of these four variables, infection did not have a significant effect. Furthermore, no effect of the infection treatment (i.e., uninfected versus infected) on mortality was detected in either the 26°C or 28°C treatment groups, and Student’s t-tests comparing the total of blood ingested up until imaginal molting revealed no significant difference between the temperatures and infection treatments at these temperatures. Therefore, the results described hereafter are composed exclusively of data from the control groups from the four temperatures evaluated.

**Fig 2:**
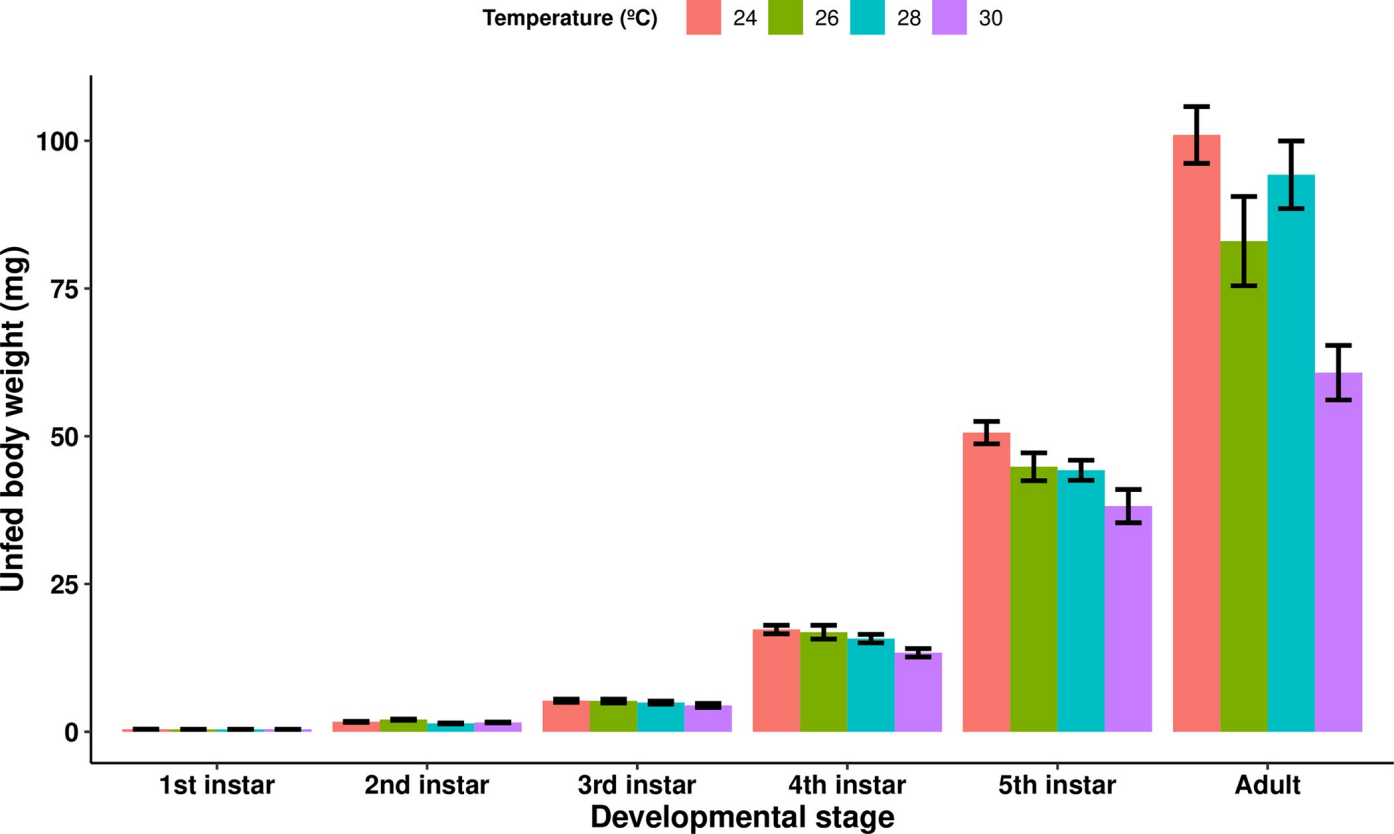
The mean unfed body weight of uninfected *Rhodnius prolixus* at different developmental stages and ambient temperatures. The unfed body weight significantly increased with increasing triatomine developmental stage (p < 2e-16, n = 120), and triatomines kept at 30°C had the lowest unfed body weight (comparison with: the 24°C treatment, p = 0.00016; the 26°C treatment, p = 0.019; and the 28°C treatment, p = 0.012; n = 120 for all comparisons). The 24°C group had a significantly higher unfed body weight compared to the 28°C group (p = 0.02905). Error bars represent the standard error of the mean.

We analyzed the dataset of all uninfected control triatomine individuals (i.e., from each temperature treatment group) simultaneously for the effects of developmental stage and temperature treatment on their unfed body weight using GEE modeling. As expected, the unfed body weight progressively increased at every developmental stage (Wald statistic = 1314.62 and p< 2e-16, n = 120), from 1.69 ± 0.047 mg 30 days after molting to the second nymphal instar to 87.8 ± 3.35 mg 30 days after imaginal molting. Furthermore, the triatomines maintained at 30°C exhibited a lower body weight than those in the other temperature treatments (compared with the 24°C treatment, Wald statistic = 14.22 and p = 0.00016; the 26°C treatment, Wald statistic = 5.54 and p = 0.019; and the 28°C treatment, Wald statistic = 6.38 and p = 0.012; n = 120 for all comparisons), and the individuals maintained at 28°C had a lower body weight than those maintained at 24°C (Wald statistic = 4.76 and p = 0.02905) (Fig 2).

**Fig 3:**
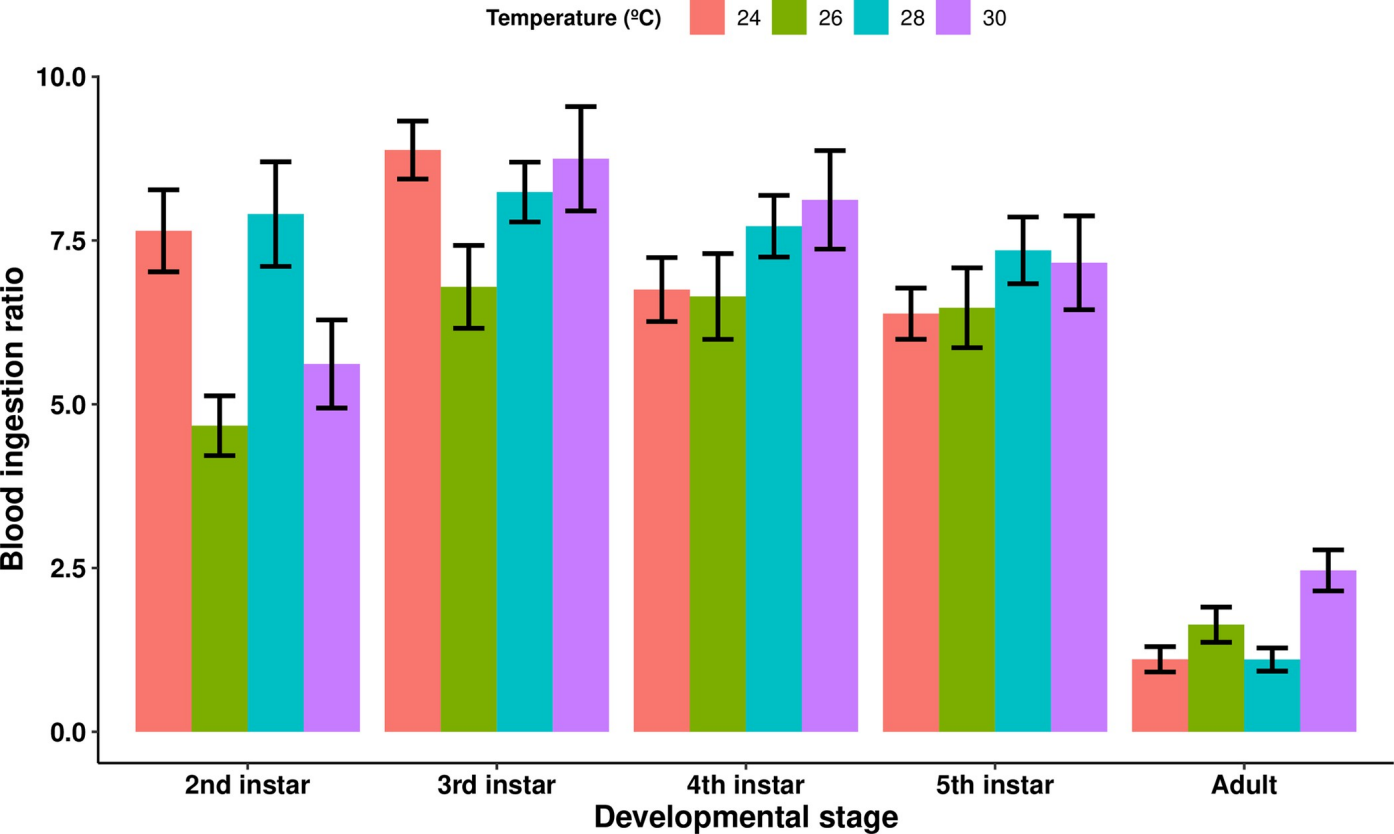
The mean blood ingestion ratio of uninfected *Rhodnius prolixus* blood-fed at different developmental stages and ambient temperatures. The blood ingestion ratio is the blood-fed body weight of each individual divided by its own unfed body weight immediately prior to blood-feeding. The blood ingestion ratio decreased with developmental stage (p = 6.9e-13, n = 111). Additionally, the triatomines kept at 26°C had the lowest blood ingestion ratio of all temperature treatments (comparison with: the 24°C treatment, p = 2.1e-05; the 28°C treatment, p = 2.5e-06; and the 30°C treatment, p = 0.00022; n = 111 for all comparisons). Error bars represent the standard error of the mean.

The GEE model analyzing the effects of developmental stage and temperature on the blood ingestion ratio of the uninfected triatomines in each temperature treatment showed that the blood ingestion ratio decreased with developmental stage (Wald statistic = 51.58 and p = 6.9e-13, n = 111). By performing a similar analysis considering only the nymphal instars (i.e., without the data from the adult stage), we observed that the blood ingestion ratio was no longer significantly different between developmental stages, which suggests that the appreciable decrease in blood ingestion ratio observed between the fifth nymphal instar and the adult stage may be the sole reason for this result. Additionally, the triatomines maintained at 26°C displayed a lower blood ingestion ratio compared to every other temperature group (comparison with: the 24°C treatment, Wald statistic = 18.11 and p = 2.1e-05; the 28°C treatment, Wald statistic = 22.2 and p = 2.5e-06; and the 30°C treatment, Wald statistic = 13.7 and p = 0.00022; n = 111 for all comparisons), with this difference being mostly observed during the second and third nymphal instars (Fig 3).

The blood ingestion ratio did not have a significant effect on the quantity of urine excreted by the triatomines after each blood meal, nor did temperature treatment, as only developmental stage had an effect in the GEE model dedicated to the quantity of urine excreted (Wald statistic = 557.966 and p < 2e-16, n = 111). By summing the volumes of each of the individual bloodmeals received up until imaginal molting, we found that the triatomines that reached the adult stage had ingested a mean ± S.E. of 449 ± 10.1 μL during their nymphal period, with the minimum quantity of blood ingested by any of the individuals who reached the adult stage being 134 μL and the maximum being 700 μL. Student’s t-tests comparing the total of blood ingested up until imaginal molting revealed no significant difference between the temperature treatments.

**Fig 4:**
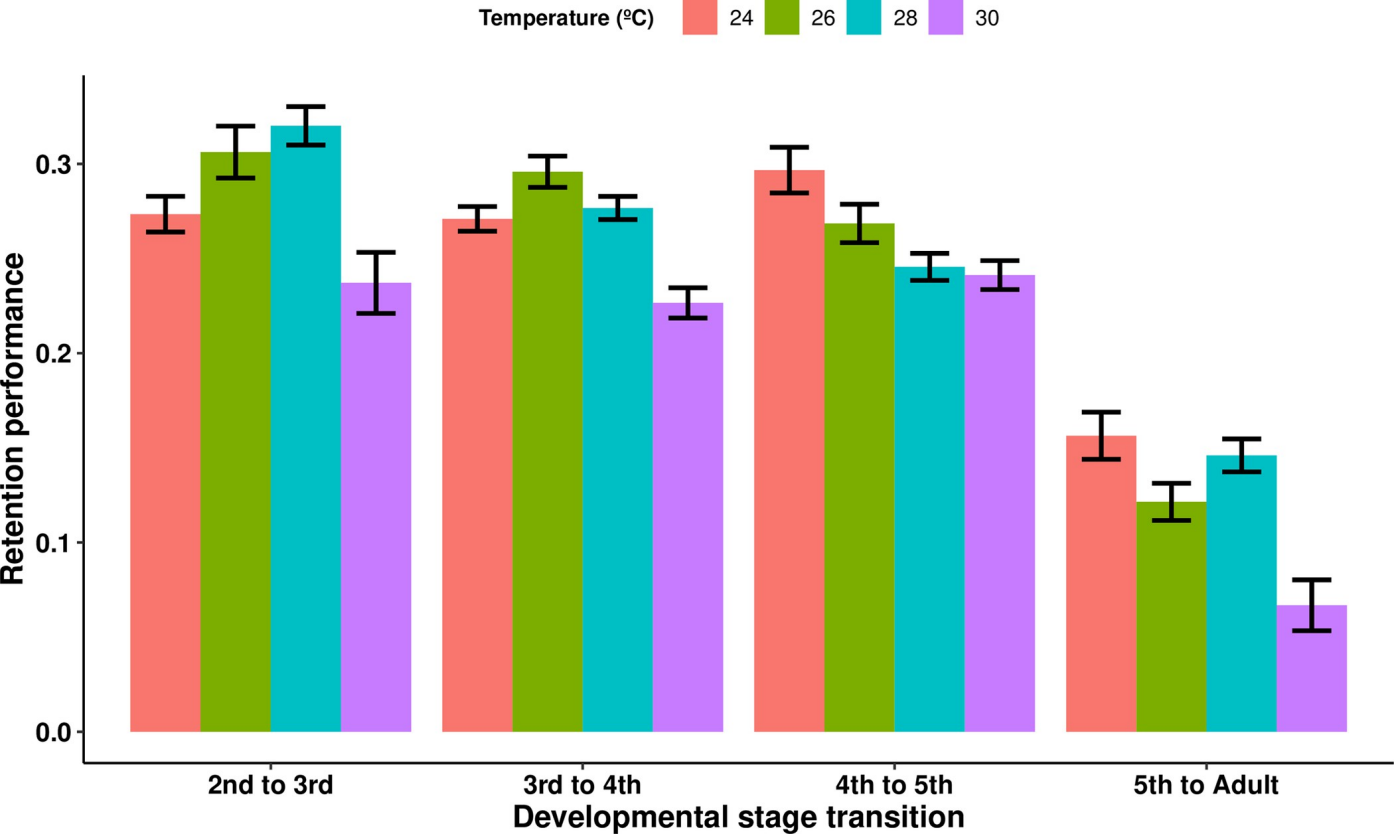
The mean retention performance of uninfected *Rhodnius prolixus* at different triatomine developmental stages and ambient temperatures. Retention performance measures the proportion of the weight of the blood meal ingested in the previous nymphal instar that is maintained as triatomine body weight in the next instar. The 30°C treatment had the lowest retention performance (comparison with: the 24°C treatment, p = 6.6e-13; the 26°C treatment, p = 3.5e-11; and the 28°C treatment, p = 1e-12; n = 94 for all comparisons). Additionally, retention performance decreased with developmental stages (p < 2e-16). Error bars represent the standard error of the mean.

The GEE model analyzing the effects of developmental stage and temperature treatment on retention performance showed that the individuals kept at 30°C had a significantly lower retention performance compared to the triatomines from every other temperature treatment (comparison with: the 24°C treatment, Wald statistic = 51.65 and p = 6.6e-13; the 26°C treatment, Wald statistic = 43.86 and p = 3.5e-11; and the 28°C treatment, Wald statistic = 50.8 and p = 1e-12; n = 94 for all comparisons). Retention performance also progressively decreased over successive developmental stages (Wald statistic = 246.49 and p < 2e-16) (Fig 4). A GEE model using only the data from the nymphal instars (i.e., excluding the data from the adult stage) shows that this decrease still exists in successive nymphal instars (Wald statistic = 5.27 and p = 0.022, n = 94).

The “molting time”, representing the duration of the period between blood feeding and subsequent molting, was analyzed by GEE modeling for effects of developmental stage and temperature treatments. As expected, the molting time progressively increased at every instar (Wald statistic = 1078 and p < 2e-16) and progressively decreased with increasing temperature, with every temperature treatment being significantly different from every other, so that the triatomines from the 24°C treatment took the longest time to molt after feeding (comparison with: the 26°C treatment, Wald statistic = 126 and p < 2e-16; the 28°C treatment, Wald statistic = 313 and p < 2e-16; and the 30°C treatment, Wald statistic = 507 and p < 2e-16). The triatomines kept at 26°C exhibited the second highest molting time (comparison with: the 28°C treatment, Wald statistic = 19.5 and p = 1e-05; the 30°C treatment, Wald statistic = 90.7 and p<2e-16), followed by the triatomines from the 28°C treatment (comparison with the 30°C treatment, Wald statistic = 33.7 and p = 6.3e-09) (Overall, n = 115), and lastly the triatomines kept at 30°C, which took 179 ± 1.63 days to reach the adult stage. For comparison, the 24°C treatment group took 215 ± 0.84 days to reach the adult stage, the 26°C treatment group took 197 ± 0.93 days, and the 28°C treatment group 189 ± 0.84 days. These durations represent the cumulative time insects took to molt after blood-feeding, added to the experimentally-imposed 30 day fasting periods between each nymphal molting and blood–feeding, which occurred at every instar (totaling 120 days over the lifespan of the insects). The 15-day period between egg hatching and the first nymphal blood-feeding was not included. As expected, these total durations to reach the adult stage differed among the temperature treatment groups when compared using Mann-Whitney U testing, with the group maintained at 30°C reaching adulthood faster than any of the other temperature treatment groups (comparison with: the 28°C treatment, p = 0.0001972 and n = 27; the 26°C treatment, p = 4.817e-05 and n = 28; and the 24°C treatment, p = 1.749e-06 and n = 33), followed by the 28°C treatment group (comparison with: the 26°C treatment, p = 0.0002738 and n = 29; the 24°C treatment, p = 3.607e-07 and n = 34), then by the 26°C treatment group (comparison with the 24°C treatment, p = 6.547e-06 and n = 35), and finally the 24°C treatment group.

**Fig 5:**
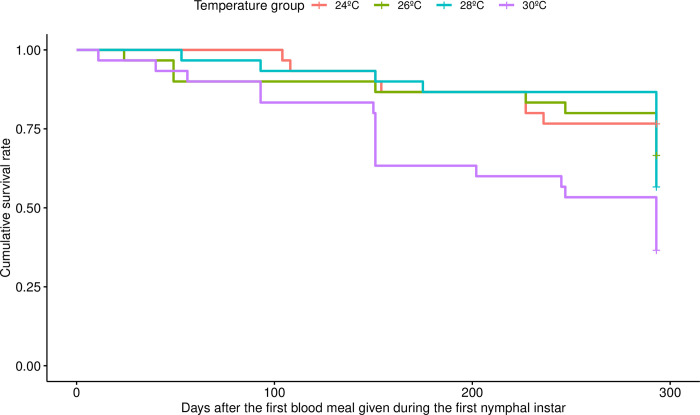
Survival rate of uninfected *Rhodnius prolixus* maintained at four different temperatures during the 293 days of the experiment. The insects kept at 30°C had lower survival than those kept at 24°C and 26°C (p = 0.003 and p = 0.02, respectively, according to the Cox proportional hazards regression model). Overall, n = 120, with n = 30 per temperature treatment group.

The survival of the triatomines was significantly affected by temperature treatment, as the individuals maintained at 30°C had significantly lower survival rates than did those in the two lowest temperature groups of 24°C (p = 0.003) and 26°C (p = 0.02) (Fig 5). By the end of the experiment, 293 days after the first alimentation at the first instar, 19 out of 30 of the uninfected triatomines from the 30°C treatment had died, while in the 24°C group, which had the least deaths of all temperature treatments, only 7 out of 30 triatomines had died. Binomial testing was used to compare the survival rate at the end of the experiment to that of a similar experiment with a shorter fasting period [11], and showed that starvation increased the mortality in the 30°C groups (p-value = 0.002584, n = 82) and 26°C groups (p-value = 0.0001461, n = 112).

## Discussion

The proportion of insects that developed a chronic infection following the infective blood meal at the first instar exhibited notable differences between the temperature treatments. At both the hottest and coldest of our temperature treatments, namely, 24°C and 30°C, we observed a percentage of insects that released parasites at any point of their life cycle of 10% and 0%, respectively. In contrast, temperatures of 26°C and 28°C both yielded chronic infection rates exceeding 90%. These findings stand in stark contrast to a prior study reporting a 100% chronic infection rate across our temperature range using the same protocol without nutritional stress [11]. At the first instar, where the infective blood meal was taken in our experiment, the insects ingest small amounts of blood (less than 3 μl). The fact that no insects placed at 30°C developed an infection could then be explained by a faster depletion of this small amount of nutrients due to the increased metabolic activity of both the insect and the parasite at higher temperatures [43], leading to the elimination of the parasites before the following meal. This could also explain why infection was lost faster in our experiment than in experiments where insects were infected at later developmental stages [15,44]. However, the fact that 24°C was the other most affected temperature treatment is puzzling. An explanation might come from the fact that at this temperature, parasite multiplication is slower than at higher temperatures in the range used in this study [6,11]. The combination of slow multiplication rate and the small amount of blood and parasites ingested may not sustain a population of parasites able to maintain the infection during the period of starvation to which the insects were subjected. Indeed, the effect of fasting on the establishment of infection was almost nonexistent at 26°C and 28°C, temperatures that are closer to the optimal and preferred temperatures of most triatomine species [28,45]. This could indicate that the use of triatomine nutrients and the multiplication rate of the parasite are appropriately adapted to the temperature range at which their interaction takes place.

The evolution of the parasite load in starved *R*. *prolixus* across developmental stages was described in the groups maintained at 26°C and 28°C, where infection rates allowed for statistical analysis. In both temperature groups, parasite load peaked during early instars—at the third instar in the 26°C group and at the fourth instar in the 28°C group—before exhibiting a substantial decline at each successive developmental stage. These results present two important differences with the evolution of parasitemia previously described in non-starved insects [11]. Firstly, the concentration of parasites at the third instar seems to be higher in starved triatomines, exceeding a thousand parasites per μL of urine in both temperature groups, than in non-starved triatomines, where this concentration rarely exceeds 100 par/μL. Secondly, the parasite load increases across developmental stages in non-starved triatomines, whereas a 30 days starvation period induced a declining trend after a peak in the early stages. These divergences suggest that in insects where chronic infection successfully occurs, a longer interval between blood meals could initially benefit the parasite population, potentially by allowing more time for the parasite to multiply and reach the developmental stage at which they are ready to be excreted. In turn, later instars take more time to molt after a blood meal, increasing the time without new nutrients in the intestine up to a detrimental amount for the parasite population.

Indeed, the amount of nutritional resources has long been suspected to be a major factor influencing the evolution and maintenance of the *T*. *cruzi* population within the intestines of triatomines. Extended periods of starvation lead to a reduction in the number of metacyclic forms in later developmental stages of *Triatoma infestans* and *Panstrongylus megistus* [4,46,47] and to an increased proportion of *Triatoma dimidiata* losing infection compared to non-starved individuals [15]. Here, we show that starvation in *R*. *prolixus* negatively affects the parasite load in chronically infected insects in later instars and that subsequent feedings are not sufficient to compensate for the losses caused by the fasting period between them. This led three positive individuals from the 26°C and 28°C temperature groups to present no parasites in the released urine at the adult stage. Additionally, in the 28°C treatment group, the triatomines with higher unfed body weight had a lower concentration of parasites in their released urine compared to lighter ones, contrasting with studies with a shorter time between blood meals, in which the heavier individuals exhibited higher parasite loads [11]. In conditions of short starvation periods, the parasite load may be correlated with the quantity of resources ingested by the vector, as the amount of available nutrients apparently allows for full development of both the insect and the parasite. Under starving conditions however, the onset of nutritional stress for the parasite could appear faster in heavier triatomines, as heavier individuals might digest the blood meal faster due to a higher metabolic requirement.

In our protocol, infection by *T*. *cruzi* did not affect any of the evaluated parameters, namely, survival rates, unfed body weight, blood ingestion ratio, retention performance, quantity of urine excreted or molting time, at the two temperatures where these comparisons were possible (i.e., 26°C and 28°C). A previous study reported negative impacts of infection on triatomine survival under similar fasting periods [6]; however, this study used an artificial feeder, leading to a considerably greater number of parasites ingested (approximately 200,000 per insect) than that of infections using an infected mammal, as in our protocol. This earlier study also evaluated the mortality rate only in the instar directly following the infective blood meal, thereby not accounting for the potential reduction in parasite virulence that could result from the observed decrease in parasite load due to starvation over the course of a chronic infection. Here, we demonstrate that infection upon ingestion of blood from an infected mouse (i.e., under conditions more closely resembling natural infections) does not sufficiently affect the resources of the insect to cause a higher mortality rate in triatomines exposed to successive starvation periods of 30 days after each molt. Interestingly, our results showed no effect of infection on the ability of the insect to maintain body weight between blood meals (i.e., retention performance), which has been observed when comparing non-starved infected and control *R*. *prolixus* [11]. Therefore, successive relatively short starvation periods may benefit the insect vector by reducing the parasite load to levels that no longer significantly affect the host’s retention performance or resistance to starvation. While we did not assess female fertility—an important fitness factor known to be influenced by infection [8,12]—our study demonstrates that under the experimental conditions used *T*. *cruzi* infection had no significant impact on key fitness components, including survival, developmental rates, and body weight. Importantly, other studies using infected mammals as the source of infection but different triatomine species and *T*. *cruzi* strains have shown pathogenic effects on starved insects, including increased mortality rates [7,15,48].

Infection has been reported to delay triatomine molting, with the presence of this effect depending on several factors, such as ambient temperature and parasite strain [6,11,21]. Although the underlying reason for this delay remains unclear, proposed hypotheses include a shift in triatomine resource allocation toward immunological responses, a decrease in the efficiency of blood nutrient intake due to competition with the parasite for resources, or the perturbation of the microbiota induced by *T*. *cruzi* [49–51]. In our study, no molting delay was observed in infected insects, despite previous findings showing delays under similar conditions with shorter fasting periods at 26°C. This discrepancy does not seem to come from a difference in the size of the parasite population, as the delay was mainly observed during the intermolt period between the fourth and fifth instars, at which point our parasite concentrations are comparable to those observed at the same temperature without nutritional stress [11]. The absence of molting delay under starvation conditions suggests that triatomines with reduced nutritional resources may prioritize allocating them toward molting to prepare faster for a potential blood meal opportunity. This observation therefore adds the nutritional status of the vector to the list of factors dictating the presence or absence of molting delays in infected triatomines.

In the uninfected control groups, the triatomines exposed to a temperature of 30°C had a lower body weight than did those in every other temperature group, and the triatomines placed at 28°C had a lower body weight than the ones placed at 24°C. This difference is not the result of a lower quantity of blood ingested by the triatomines, as the blood ingestion ratio of the triatomines in the higher temperature groups was not lower than that of those in the lower temperature groups. In fact, our results showed that the insects placed at 26°C had the lowest blood ingestion ratio of all temperature groups, and although the reason behind this effect are unclear, their unfed body weight was still higher than that of the 30°C temperature group. The lower body weight at high temperatures, rather, seems to be due to a decrease in retention performance, as this parameter was significantly reduced in the insects kept at 30°C. We previously described this difference in retention performance across temperature treatments, although this effect was not sufficient to cause a difference in body weight between temperature groups when *R*. *prolixus* was subjected to a 15-day fasting period [11]. This suggests that the minimum fasting time necessary for the body weight of *R*. *prolixus* to be significantly affected by high temperatures lies between 15 and 30 days.

Therefore, in part due to the combined effects of greater water loss stress, lower body weight and increased metabolic rate, the triatomines in the 30°C group had a substantially lower survival rate than those in the other temperature treatments. In addition, the mortality rate observed in insects kept at 30°C and 26°C by the end of our experiment was considerably higher than in a similar protocol with shorter fasting periods [11]. Previously, we emphasized the importance of considering the contradictory effects of increased ambient temperature, with higher mortality rates but accelerated life cycle, when modeling the effects of temperature on *R*. *prolixus* populations [11]. In the present study, we introduced the nutritional condition of the vector as a critical parameter influencing this dynamic, affecting both mortality rates and life cycle, depending on ambient temperature. This suggests that the availability of hosts will be a key factor in determining how global warming will affect the populations of this vector.

In conclusion, we showed that fasting time could have important consequences for the life cycle of *R*. *prolixus*, the onset and progression of *T*. *cruzi* infection, and the effects of temperature on these organisms and their interaction. We demonstrated that the minimal fasting time for starvation to be detrimental to the *T*. *cruzi* population lies between 15 and 30 days after molting in the later developmental stages of *R*. *prolixus*. Our results, along with previous research [11], suggest that above 24°C, the nutritional state of the vector is one of the most important factors dictating the *T*. *cruzi* population dynamics within *R*. *prolixus*, although particularly low and high temperatures strongly affected the establishment of infection in early instars. Conversely, 30-day starvation periods impaired *R*. *prolixus* survival and weight retention only at relatively high temperatures, being well tolerated at the preferred temperatures of the insect [28], suggesting that ambient temperature is one of the governing factors in the life cycle of this ectotherm. Alongside global warming, the short- and long-term effects of climate change on the ecology of this host-pathogen system are highly multifactorial, as changes in seasonality, host diversity and availability, natural disasters, increased CO_2_ concentrations [31–33,52], as well as insect adaptation and thermopreference [18,28], can all have important interplays in shaping the spatiotemporal evolution of the epidemiology of Chagas disease. We also acknowledge the limited genetic variability inherent to laboratory colonies, potentially affecting the life-cycle of the insect and its relationship with the parasite. To confirm the broader applicability of these results, further validations involving sylvatic populations of *R*. *prolixus* are warranted. Nevertheless, our study highlights the importance of considering the nutrition of the vector when modeling the potential effects of climate change on Chagas disease, and presents new hypotheses for future research on the interaction between vectors and parasites in a rapidly changing world.

## Supporting information

S1 Statistical models The structures and results of the GEE and linear mixed effects models created and discussed in this study. (DOCX)

S1 Dataset The complete dataset produced and analyzed in this study. (XLSX)

S1 Fig Graphical representation of the parasite concentration in excreted urine at several life stages at 26°C and 28°C, in insects under a 15 or 30 days fasting period. (TIF)
