# Supplementary material for: Effects of fasting on the interplay between temperature and Trypanosoma cruzi infection on the life cycle of the Chagas disease vector Rhodnius prolixus
Source: PLoS Negl Trop Dis. 2024 Nov 11;18(11):e0012665. doi: 10.1371/journal.pntd.0012665 (PMC11581405; doi:10.1371/journal.pntd.0012665)
Supplement: S1 Statistical models — (DOCX) [file pntd.0012665.s001.docx]

Statistical models

All formulas and results presented here were created and obtained using the *geepack* (1), *gee* (2), and nlme (3,4) packages in R (version 4.2.1)(5).

1. GEE model 1: Differences in excreted urine volume depending on blood ingestion ratio, temperature and developmental stage:

GEE formula:

geeglm(formula = urine ~ Developmental Stage + Temperature * Blood Ingestion Ratio, family = poisson, data = Control Nymfas, id = Data, corstr = "exchangeable")

| Term | Estimate | Std. Err | Wald | Pr (>\|W\|) |
| --- | --- | --- | --- | --- |
| (Intercept) | -1.854672 | 0.363018 | 26.1 | 3.2e-07 *** |
| Developmental Stage | 1.104717 | 0.046768 | 557.97 | < 2e-16 *** |
| Temperature26 | -0.131552 | 0.43218 | 0.09 | 0.76 |
| Temperature28 | -0.207816 | 0.435207 | 0.23 | 0.63 |
| Temperature30 | -0.073607 | 0.461712 | 0.03 | 0.87 |
| timesweighteaten | 0.028035 | 0.039123 | 0.51 | 0.47 |
| Temperature26:timesweighteaten | -0.042443 | 0.053612 | 0.63 | 0.43 |
| Temperature28:timesweighteaten | 0.000566 | 0.054883 | 0 | 0.99 |
| Temperature30:timesweighteaten | -0.053776 | 0.057962 | 0.86 | 0.35 |

Signif. codes: 0 ‘***’ 0.001 ‘**’ 0.01 ‘*’ 0.05 ‘.’ 0.1 ‘ ’ 1

Parameters:

| Estimated Scale Parameters | Estimate | Std. Err |
| --- | --- | --- |
|  | 7.08 | 0.564 |
| Estimated Correlation Parameters | Estimate | Std. Err |
| alpha | 0.138 | 0.0565 |
| Number of clusters: 111 Maximum cluster size: 4 | | |
| Model Fit Statistics | QIC | QICu |
|  | -19891 | -19909 |

1. GEE model 2: differences in Blood Ingestion Ratio depending on temperature and developmental stage in individuals from the control group:

GEE formula:

geeglm(formula = BloodIngestionRatio ~ DevelopmentalStage + Temperature, family = gaussian, data = Controls, id = Data, corstr = "exchangeable")

| **Term** | **Estimate** | **Std.err** | **Wald** | **Pr(>\|W\|)** |
| --- | --- | --- | --- | --- |
| (Intercept) | 9.7474 | 0.45 | 469.21 | < 2e-16 *** |
| Developmental Stage | -0.7732 | 0.1077 | 51.58 | 6.9e-13 *** |
| Temperature26 | -1.3825 | 0.3248 | 18.11 | 2.1e-05 *** |
| Temperature28 | 0.0948 | 0.3051 | 0.1 | 0.76 |
| Temperature30 | -0.0503 | 0.365 | 0.02 | 0.89 |

Signif. codes: 0 ‘***’ 0.001 ‘**’ 0.01 ‘*’ 0.05 ‘.’ 0.1 ‘ ’ 1

| **Estimated Scale Parameters** | |  |
| --- | --- | --- |
| **Term** | **Estimate** | **Std.err** |
| (Intercept) | 9.91 | 0.691 |

| **Estimated Correlation Parameters** | |  |
| --- | --- | --- |
| **Parameter** | **Estimate** | **Std.err** |
| alpha | -0.0993 | 0.0263 |

| Number of clusters | 111 |
| --- | --- |
| Maximum cluster size | 5 |

| **Model Fit Statistics** |  |
| --- | --- |
| **Metric** | **Value** |
| QIC | 4120.59 |
| QICu | 4124.12 |
| Quasi Lik | -2057.06 |
| CIC | 3.24 |
| Params | 5 |
| QICC | 4120.8 |

1. GEE model 2: differences in molting time depending on temperature and developmental stage in individuals from the control group:

GEE formula:

geeglm(formula = MoltingTime ~ DevelopmentalStage + Temperature, family = poisson,

data = Controls, id = Data, corstr = "exchangeable")

| Term | Estimate | Std.err | Wald | Pr(>\|W\|) |
| --- | --- | --- | --- | --- |
| (Intercept) | 2.2608 | 0.02091 | 11688 | <2e-16*** |
| Developmental stage | 0.21373 | 0.00651 | 1078 | <2e-16*** |
| Temperature26 | -0.21755 | 0.01938 | 126 | <2e-16*** |
| Temperature28 | -0.32631 | 0.01843 | 313 | <2e-16*** |
| Temperature30 | -0.48999 | 0.02175 | 507 | <2e-16*** |

**Estimated Scale Parameters**

| Parameter | Estimate | Std.err |
| --- | --- | --- |
| (Intercept) | 0.62 | 0.076 |

**Estimated Correlation Parameters**

| Parameter | Estimate | Std.err |
| --- | --- | --- |
| alpha | -0.131 | 0.0312 |

Number of clusters: 115 Maximum cluster size: 5

**Model Fit Statistics**

| Metric | Value |
| --- | --- |
| QIC | -22361.8 |
| QICu | -22358.33 |
| Quasi Likelihood | 11184.16 |
| CIC | 3.27 |
| Number of parameters | 5 |
| QICC | -22361.6 |

1. GEE model 3: differences in Unfed weight depending on temperature and developmental stage in individuals from the control group:

GEE formula:

geeglm(formula = UnfedWeight ~ DevelopmentalStage + Temperature, family = Gamma, data = Controls, id = Data, corstr = "exchangeable")

| Variable | Estimate | Std.err | Wald | Pr(>\|W\|) |
| --- | --- | --- | --- | --- |
| (Intercept) | 0.307164 | 0.008253 | 1385.13 | <2e-16 *** |
| Developmental Stage | -0.050254 | 0.001386 | 1314.62 | <2e-16 *** |
| Temperature26 | 0.001903 | 0.001184 | 2.58 | 0.10798 |
| Temperature28 | 0.001926 | 0.000883 | 4.76 | 0.02905 * |
| Temperature30 | 0.006619 | 0.001755 | 14.22 | 0.00016 *** |

**Estimated Scale Parameters**

| Parameter | Estimate | Std.err |
| --- | --- | --- |
| (Intercept) | 0.821 | 0.0326 |

**Estimated Correlation Parameters**

| Parameter | Estimate | Std.err |
| --- | --- | --- |
| alpha | -0.131 | 0.00919 |

Number of clusters: 120 Maximum cluster size: 6

**Model Fit Statistics**

| Metric | Value |
| --- | --- |
| QIC | 1363.57 |
| QICu | 1371.35 |
| Quasi Likelihood | -680.68 |
| CIC | 1.11 |
| Number of parameters | 5 |
| QICC | 1363.72 |

1. GEE model 3: differences in Retention performance depending on temperature and developmental stage in individuals from the control group:

GEE formula:

geeglm(formula = Retention performance ~ DevelopmentalStage + Temperature, family = gaussian, data = Control, id = Data, corstr = "exchangeable")

| Variable | Estimate | Std.err | Wald | Pr(>\|W\|) |
| --- | --- | --- | --- | --- |
| (Intercept) | 0.453992 | 0.012577 | 1303.05 | <2e-16 *** |
| Developmental Stage | -0.045595 | 0.002904 | 246.49 | <2e-16 *** |
| Temperature26 | -0.001232 | 0.00681 | 0.03 | 0.86 |
| Temperature28 | -0.000689 | 0.006324 | 0.01 | 0.91 |
| Temperature30 | -0.052596 | 0.007318 | 51.65 | 6.6e-13 *** |

**Estimated Scale Parameters**

| Parameter | Estimate | Std.err |
| --- | --- | --- |
| (Intercept) | 0.00343 | 0.000401 |

**Estimated Correlation Parameters**

| Parameter | Estimate | Std.err |
| --- | --- | --- |
| alpha | -0.134 | 0.0283 |

Number of clusters: 94 Maximum cluster size: 4

**Model Fit Statistics**

| Metric | Value |
| --- | --- |
| QIC | 7.61 |
| QICu | 11.12 |
| Quasi Likelihood | -0.56 |
| CIC | 3.25 |
| Number of parameters | 5 |
| QICC | 7.87 |

1. LME model 1: Differences in parasite concentrations depending on Developmental stage in insects from the 28ºC treatment

lme(parasiteconcentration ~ DevelopmentalStage, random = ~1 | Data/DevelopmentalStage, data = droplevels(subset(Infected,Temperature=="28")),na.action = na.exclude)

| **Model Information** |  |
| --- | --- |
| **Metric** | **Value** |
| AIC | 473 |
| BIC | 481 |
| logLik | -230 |

| **Random effect: Formula: ~1\| Data** |  |  |
| --- | --- | --- |
|  | (Intercept) | Residual |
| StdDev: | 305 | 523 |

| **Fixed Effects**: parasiteconcentration ~ DevelopmentalStage | |  |  |  |  |
| --- | --- | --- | --- | --- | --- |
| **Effect** | **Value** | **Std.Error** | **DF** | **t-value** | **p-value** |
| (Intercept) | 581 | 225 | 19 | 2.585 | 0.0182 |
| age4 | 694 | 285 | 10 | 2.436 | 0.0351 |
| age5 | 9 | 268 | 10 | 0.034 | 0.9735 |
| age6 | -511 | 398 | 10 | -1.284 | 0.2282 |

| **Correlation** | **(Intercept)** | **age4** | **age5** |
| --- | --- | --- | --- |
| age4 | -0.722 |  |  |
| age5 | -0.802 | 0.643 |  |
| age6 | -0.524 | 0.406 | 0.458 |

| **Standardized Within-Group Residuals** | |  |  |  |
| --- | --- | --- | --- | --- |
| **Min** | **Q1** | **Med** | **Q3** | **Max** |
| -1.429 | -0.54 | -0.17 | 0.255 | 1.831 |
| **Number of Observations**: 33 **Number of Groups**: 20 | | |  |  |

1. LME model 2: Differences in parasite concentrations depending on Developmental stage in insects from the 26ºC treatment

lme(parasiteconcentration ~ DevelopmentalStage, random = ~1 | Data/DevelopmentalStage, data = droplevels(subset(Infected, Temperature=="26")),na.action = na.exclude)

| **Model Information** |  |
| --- | --- |
| **Metric** | **Value** |
| AIC | 392 |
| BIC | 399 |
| logLik | -190 |

| **Random Effects** |  |
| --- | --- |
| **Component** | **StdDev** |
| Intercept | 0.0575 |
| Residual | 564 |

| **Fixed Effects** |  |  |  |  |  |
| --- | --- | --- | --- | --- | --- |
| **Effect** | **Value** | **Std.Error** | **DF** | **t-value** | **p-value** |
| (Intercept) | 1185 | 213 | 18 | 5.56 | 0 |
| age4 | -505 | 302 | 6 | -1.67 | 0.1452 |
| age5 | -785 | 278 | 6 | -2.82 | 0.0302 |
| age6 | -969 | 354 | 6 | -2.74 | 0.0337 |

| **Correlation** |  |  |  |  |
| --- | --- | --- | --- | --- |
| **Term** | **(Intercept)** | **age4** | **age5** | **age6** |
| (Intercept) | 1 | -0.707 | -0.767 | -0.603 |
| age4 | -0.707 | 1 | 0.542 | 0.426 |
| age5 | -0.767 | 0.542 | 1 | 0.462 |
| age6 | -0.603 | 0.426 | 0.462 | 1 |

| **Standardized Within-Group Residuals** | |
| --- | --- |
| **Statistic** | **Value** |
| Min | -1.413 |
| Q1 | -0.457 |
| Med | -0.192 |
| Q3 | 0.258 |
| Max | 3.702 |

| Number of Observations | 28 |
| --- | --- |
| Number of Groups | 19 |

1. LME model 3: Differences in parasite concentrations depending on Developmental stage and fasting time in insects from the 26ºC treatment

lme(parasiteconcentration ~ DevelopmentalStage*Fasting, random = ~1 | Data/DevelopmentalStage, data = droplevels(subset(Infected, Temperature=="26")), na.action = na.exclude)

| **Model Information** |  |
| --- | --- |
| **Metric** | **Value** |
| AIC | 1086 |
| BIC | 1102 |
| logLik | -536 |

| **Random Effects** |  |
| --- | --- |
| **Component** | **StdDev** |
| Intercept | 651 |
| Residual | 3.75 |

| **Parameter** | **Value** | **Std.Error** | **DF** | **t-value** | **p-value** |
| --- | --- | --- | --- | --- | --- |
| (Intercept) | -1246 | 448 | 39 | -2.78 | 0.0083 |
| age | 449 | 103 | 28 | 4.37 | 0.0002 |
| WithFasting | 3358 | 708 | 39 | 4.75 | 0.00001 |
| Age*WithFasting | -784 | 159 | 28 | -4.93 | 0.00001 |

| **Correlation** | **(Intercept)** | **age** | **WithFasting** |
| --- | --- | --- | --- |
| age | -0.975 |  |  |
| WithFasting | -0.633 | 0.617 |  |
| Age*WithFasting | 0.629 | -0.645 | -0.975 |

| **Residuals** | **Value** |
| --- | --- |
| Min | -0.00832 |
| Q1 | -0.003213 |
| Median | -0.000842 |
| Q3 | 0.001865 |
| Max | 0.022343 |

| **Number of Observations** | **71** |
| --- | --- |
| Number of Groups | 41 |

1. LME model 4: Differences in parasite concentrations depending on Developmental stage and fasting time in insects from the 28ºC treatment

lme(parasiteconcentration ~ DevelopmentalStage*Fasting, random = ~1 | Data/DevelopmentalStage, data = droplevels(subset(Infected, Temperature=="28")), na.action = na.exclude)

| **Model Information** |  |
| --- | --- |
| **Metric** | **Value** |
| AIC | 1228 |
| BIC | 1244 |
| logLik | -607 |

| **Random Effects** |  |
| --- | --- |
| **Component** | **StdDev** |
| Intercept | 719 |
| Residual | 6.3 |

| **Parameter** | **Value** | **Std.Error** | **DF** | **t-value** | **p-value** |
| --- | --- | --- | --- | --- | --- |
| (Intercept) | -712 | 443 | 39 | -1.61 | 0.1159 |
| age | 337 | 100 | 39 | 3.36 | 0.0017 |
| WithFasting | 2195 | 755 | 36 | 2.91 | 0.0062 |
| Age*WithFasting | -506 | 169 | 39 | -2.99 | 0.0048 |

| **Correlation** | **(Intercept)** | **age** | **WithFasting** |
| --- | --- | --- | --- |
| age | -0.971 |  |  |
| WithFasting | -0.587 | 0.569 |  |
| Age*WithFasting | 0.576 | -0.593 | -0.976 |

| **Residuals** | **Value** |
| --- | --- |
| Min | -0.016 |
| Q1 | -0.00506 |
| Median | -0.00253 |
| Q3 | 0.00369 |
| Max | 0.03122 |

| **Number of Observations** | **79** |
| --- | --- |
| Number of Groups | 38 |

Tools used:

1. Halekoh U, Højsgaard S, Yan J. The *R* Package **geepack** for Generalized Estimating Equations. J Stat Soft [Internet]. 2006 [cited 2023 Feb 13];15(2). Available from: http://www.jstatsoft.org/v15/i02/

2. Carey VJ. Carey VJ (2022). _gee: Generalized Estimation Equation Solver_. R package version 4.13-23, <https://CRAN.R-project.org/package=gee> [Internet]. 2022. Available from: https://CRAN.R-project.org/package=gee

3. Mixed-Effects Models in S and S-PLUS [Internet]. New York: Springer-Verlag; 2000 [cited 2024 Mar 5]. (Statistics and Computing). Available from: http://link.springer.com/10.1007/b98882

4. Pinheiro J, Bates D, R Core Team. nlme: Linear and Nonlinear Mixed Effects Models [Internet]. 2023. Available from: https://CRAN.R-project.org/package=nlme

5. R Core Team. R Core Team (2022). R: A language and environment for statistical computing. R Foundation for Statistical Computing, Vienna, Austria. URL https://www.R-project.org/. 2022.
