# Supplementary figures and images for: Effects of fasting on the interplay between temperature and Trypanosoma cruzi infection on the life cycle of the Chagas disease vector Rhodnius prolixus
Source: PLoS Negl Trop Dis. 2024 Nov 11;18(11):e0012665. doi: 10.1371/journal.pntd.0012665 (PMC11581405; doi:10.1371/journal.pntd.0012665)

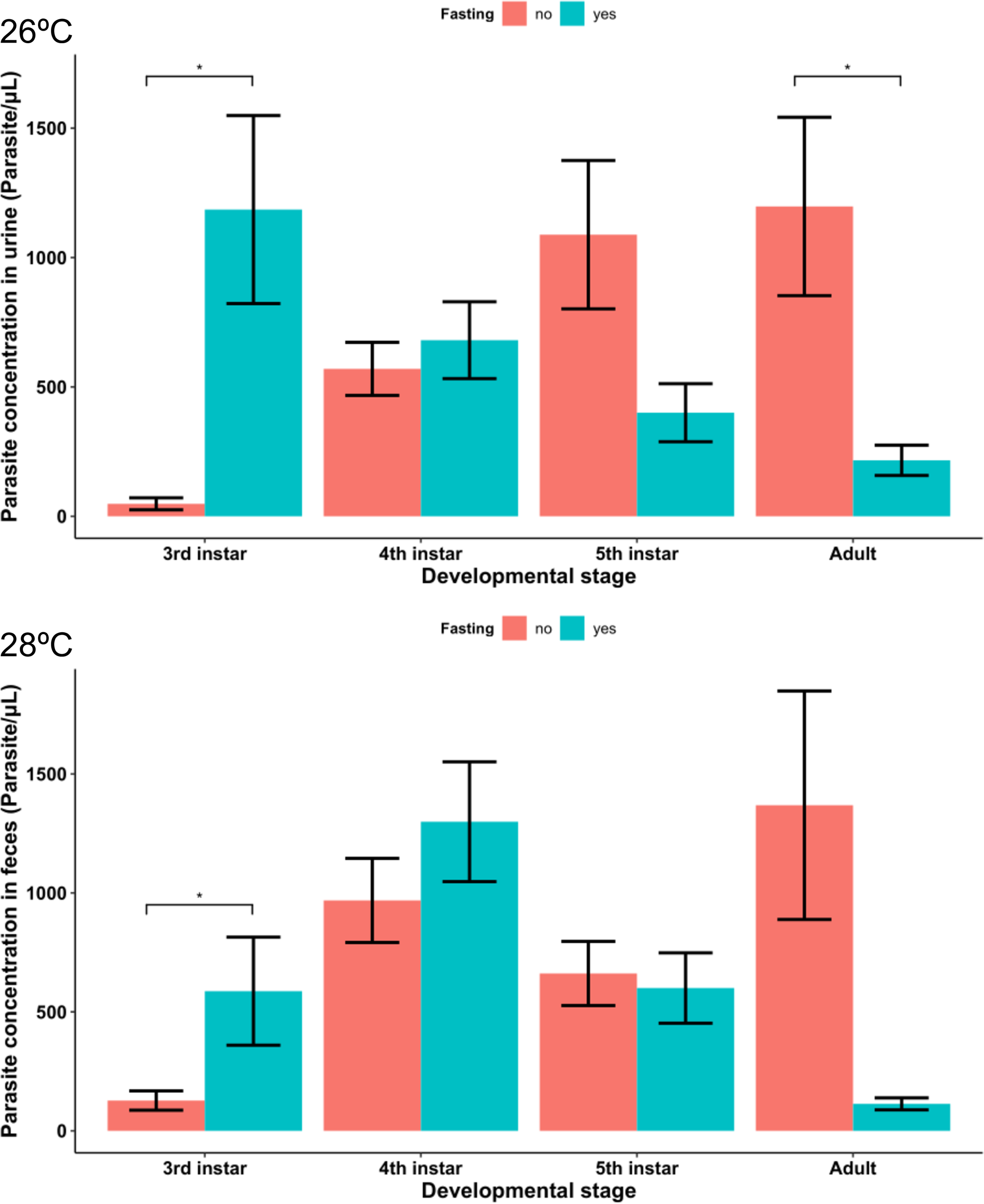

Supplement: S1 Fig — (TIF) [file pntd.0012665.s003.tif]
